# Overtraining Syndrome as a Risk Factor for Bone Stress Injuries among Paralympic Athletes

**DOI:** 10.3390/medicina60010052

**Published:** 2023-12-27

**Authors:** Tomislav Madzar, Tonci Masina, Roko Zaja, Snjezana Kastelan, Jasna Pucarin Cvetkovic, Hana Brborovic, Matija Dvorski, Boris Kirin, Andreja Vukasovic Barisic, Ivan Cehok, Milan Milosevic

**Affiliations:** 1 Polyclinic Life, Trpinjska 5, 10000 Zagreb, Croatia; tomislav_madzar@yahoo.com; 2 School of Medicine, University of Zagreb, Salata 3, 10000 Zagreb, Croatia; tonci.masina@mef.hr (T.M.); roko.zaja@snz.hr (R.Z.); snjezana.kastelan@mef.hr (S.K.); jpucarin@snz.hr (J.P.C.); hana.brborovic@snz.hr (H.B.); matija.dvorski@snz.hr (M.D.); 3 Croatian Paralympic Committee, Savska Cesta 137, 10000 Zagreb, Croatia; boris.kirin@bj.ht.hr (B.K.); andreja.vukasovic@yahoo.com (A.V.B.); 4 General County Hospital Bjelovar, Antuna Mihanovica 8, 43000 Bjelovar, Croatia; 5 Department of Nursing, University North, 104 Brigade 3, 42000 Varazdin, Croatia; icehok@unin.hr

**Keywords:** overtraining syndrome, risk factor, bone stress injuries, paralympic athletes

## Abstract

*Background and Objectives:* In this review, we have explored the relationship between overtraining syndrome (OTS) and bone stress injuries among paralympic athletes. OTS is a complex condition that arises from an imbalance between training volume, nutrition, and recovery time, leading to significant negative effects on paralympic athlete’s performance and overall well-being. On the other hand, bone stress injuries occur when abnormal and repetitive loading is applied to normal bone, resulting in microdamage accumulation and potential. The prevalence of overtraining syndrome and bone stress injuries among athletes highlights the need for a better understanding of their relationship and implications for prevention and management strategies. *Methods:* A literature review from the PubMed, Web of Science, and Google Scholar databases including the MeSH keywords “overtraining syndrome”, “bone”, and “paralympic athletes”. *Results:* Studies have consistently shown that athletes engaged in endurance sports are particularly susceptible to overtraining syndrome. The multifactorial nature of this condition involves not only physical factors, but also psychological and environmental determinants. In addition, the diagnosis and management of OTS and bone stress injuries present challenges in clinical practice. *Conclusions:* Currently, there are no definitive biochemical markers for overtraining syndrome. The diagnosis is based on a combination of subjective measures such as questionnaires, symptoms checklists, and objective biomarkers, including hormone levels, inflammatory markers, and imaging studies. However, these diagnostic approaches have limitations regarding their specificity and sensitivity.

## 1. Introduction

Overtraining syndrome (OTS) and associated bone stress injuries are significant concerns among athletes, with a subsequent profound impact on their performance and overall health. OTS refers to a state of chronic fatigue and decreased performance resulting from an imbalance between training load, nutrition, and recovery time [1]. On the other hand, bone stress injuries are a common type of overuse injury characterized by the accumulation of microdamage in bone tissue due to repetitive loading [2].

The prevalence of OTS and bone stress injuries among athletes is a growing concern in the field of sports medicine. Currently, varying rates of overtraining syndrome have been reported, even up to 30% among young athletes. Additionally, around 10–20% of all sports medicine injuries were stress fractures. These conditions can have significant consequences for athletes, leading to decreased performance, prolonged recovery periods, and even long-term health implications [1,2].

OTS can also affect paralympic athletes just as it can impact athletes in other sports. Paralympic athletes face unique challenges due to their disabilities, but the principles of overtraining and risk factors remain largely the same. Paralympic athletes often participate in intensive training programs to enhance their physical performance and excel in their respective sports. However, when the training load exceeds the body’s ability to recover, OTS can occur. Diagnosing OTS can be challenging as it involves a combination of subjective and objective measures. There is no specific medical test that can definitively diagnose OTS. Instead, healthcare professionals rely on a comprehensive evaluation of an athlete’s symptoms, training history, and performance changes.

Detailed understanding of the relationship between OTS and bone stress injuries is crucial for the development of effective prevention and management strategies. The interplay between training load, energy availability, hormonal imbalances, genetic factors, neuromuscular control, biomechanics, inflammatory markers, and psychosocial factors has been explored as they are potential contributors to the development of both conditions [3,4,5,6]. In this review article, our aim was to contribute to the body of knowledge surrounding OTS as a risk factor for bone stress injuries among paralympic athletes by encompassing risk factors, mechanism of action explanations, diagnostic possibilities, and prevention strategies. Due to the nature and classification of paralympic athletes there are high possibilities of overtraining in disability compensation compared to non-paralympic athletes.

## 2. Methods

A literature review from the PubMed, Web of Science, and Google Scholar databases including the MeSH (Medical Subject Headings) keywords “overtraining syndrome”, “bone”, and “paralympic athletes” has been made. Out of 37 found papers, we included 28 papers in the English language with full-text availability covering the last 20 years (from 2003 to 2023).

## 3. Risk Factors and their Mechanism of Action

Age has been identified as an intrinsic factor that may influence an athlete’s risk of developing OTS. Younger athletes are often more susceptible due to their higher training intensities and inadequate recovery periods [1]. However, the role of age as a risk factor for OTS remains controversial. While some studies suggested that younger athletes are at higher risk [7], others did not report a significant association between age and the incidence of OTS [8].

Gender is another important intrinsic factor that appears to influence the prevalence of OTS among athletes. Females have been found to be at a higher risk compared to males [1]. Hormonal fluctuations throughout the menstrual cycle may contribute to this increased vulnerability among women. For example, estrogen levels during certain phases of the menstrual cycle have been associated with decreased exercise performance and increased fatigue [9]. Armento et al. investigated gender differences in physiological responses to training load among endurance runners and found that female athletes exhibited different patterns of hormonal responses compared to males during periods of high training load. This highlights the importance of considering gender-specific factors when studying overtraining syndrome and bone stress injuries [4].

A medical history of previous injury has also been suggested as a potential risk factor for developing OTS. Athletes with previous injuries may have altered movement patterns or imbalances that can contribute to overuse and overtraining [1]. However, there is limited research that specifically examines the relationship between previous injury history and OTS incidence.

Extrinsic factors related to training volume/intensity, recovery periods, and nutrition are crucial contributors to the risk of developing OTS in an athlete. A high training volume/intensity without adequate recovery periods is a common cause of OTS [1]. Numerous studies have emphasized the importance of periodization in training programs by incorporating appropriate rest periods [10,11].

OTS is a complex condition influenced by various intrinsic and extrinsic factors that can increase an athlete’s susceptibility to its development.

### 3.1. Inflammatory Cytokines

In recent years, there has been an emerging interest in investigating the role of inflammatory markers in the pathogenesis of OTS and bone stress injuries among athletes [3,5]. Inflammation plays a crucial role in tissue repair processes, but excessive or prolonged inflammation may contribute to tissue damage, highlighting the potential role of inflammatory cytokines, such as interleukin-6 (IL-6), tumor necrosis factor-alpha (TNF-α), and C-reactive protein (CRP), in OTS. Studies have proposed that elevated levels of these markers may contribute to the development of fatigue, muscle damage, and impaired immune function commonly observed in athletes with OTS. Schwellnus et al. discussed the possible involvement of inflammation in bone stress injuries among athletes. They suggested that pro-inflammatory cytokines and chemokines released during repetitive loading can lead to an imbalance between bone resorption and formation processes, ultimately increasing the risk of stress fractures [3]. Furthermore, it was thoroughly described how IL-6 and other pro-inflammatory cytokines stimulate osteoblasts to express receptor activator of nuclear factor kappa-Β ligand (RANKL), which then binds to receptor activator of nuclear factor kappa-Β (RANK) in osteoclasts, leading to the stimulation of bone resorption [12]. Although these studies provided information on the potential role of inflammation in both OTS and bone stress injuries, further research is needed to elucidate the underlying mechanisms and establish causality.

### 3.2. Genetic Factors

Genetic factors have also been implicated as potential contributors to individual susceptibility to OTS and bone stress injuries. Tenforde et al. discussed the importance of neuromuscular control in preventing excessive loading on bones and highlighted the potential benefits of targeted strength training programs to improve neuromuscular function [5]. Furthermore, investigating potential genetic factors that influence susceptibility to OTS could provide valuable information on individual variations in response to training loads. Understanding genetic predispositions may help identify athletes at higher risk for developing OTS or bone stress injuries.

Several genetic variations have been investigated for their potential association with overtraining-related outcomes such as fatigue resistance, muscle damage markers, and inflammatory responses. For example, polymorphisms in genes related to collagen synthesis, such as COL5A1 and COL1A1, have been studied in the context of OTS and bone stress injuries. However, the specific genetic factors that contribute to the risk of OTS are still not well understood, and more research is required to elucidate their role [13,14].

### 3.3. Nutritional Deficiencies and Energy Availability

Nutritional deficiencies or imbalances can significantly impact an athlete’s susceptibility to both OTS and bone stress injuries. Inadequate energy intake, particularly low energy availability (LEA), has emerged as a significant risk factor for the development of both conditions [15]. LEA occurs when an athlete’s energy intake does not meet the energy demands of training and normal physiological functions, leading to negative consequences on various body systems including hormonal regulation, immune function, metabolic processes, and bone health [15,16]. Studies have shown that athletes with LEA are at increased risk for developing both OTS and bone stress injuries [4,5]. Cupka and Sedliak reviewed the impact of low energy availability on the performance and testosterone levels of male endurance athletes. This metabolic disturbance can contribute to the onset of OTS symptoms [17]. Energy availability refers to the amount of dietary energy intake available for physiological functions after accounting for energy expended during exercise. It is influenced by factors such as caloric intake, exercise expenditure, thermoregulation, growth, repair processes, and reproductive function [15]. The balance between energy intake and expenditure is crucial to maintaining optimal health and performance among athletes. This imbalance can have significant consequences for various physiological systems in the body. In the context of athletics, low energy availability often arises from intentional or unintentional restrictions in food intake due to concerns about body weight or composition [16].

The influence of energy availability, a key component in the development of OTS, has also been explored in relation to bone stress injuries. Low energy availability can lead to a condition known as relative energy deficiency in sport (RED-S), characterized by hormonal imbalances, impaired bone health, and increased risk of injury [5]. Mountjoy et al. proposed that RED-S encompasses a range of adverse health outcomes resulting from inadequate energy availability, including suppression of metabolic rates, menstrual disturbances in women, decreased testosterone levels in men, impaired bone health, cardiovascular dysfunction, immunological impairments, psychological disturbances, gastrointestinal problems, hematological abnormalities, and impaired growth and development in adolescents [15]. Several studies have examined the association between RED-S/LEA and an increased risk of bone stress injuries among athletes. A narrative review by Hamstra-Wright et al. highlighted the importance of a holistic approach to monitoring training load in relation to bone stress injuries. The authors emphasized the need for a personalized assessment that considers individual risk factors and cumulative risks associated with the training load capacity [18].

The relationship between low energy availability and bone health has been extensively studied. LEA can disrupt hormonal balance, leading to menstrual irregularities in female athletes and decreased testosterone levels in male athletes [16]. These hormonal changes can have detrimental effects on bone health, resulting in decreased bone mineral density and increased susceptibility to fractures [15]. In a study by Tenforde et al., female college distance runners with LEA were found to have significantly lower bone mineral density in the lumbar spine compared to their counterparts with normal EA. Furthermore, LEA can affect bone remodeling processes by affecting both osteoblasts and osteoclast activity [5]. Armento et al. discussed how LEA may lead to decreased osteoblast function through alterations in insulin-like growth factor-1 (IGF-1), estrogen levels, leptin signaling pathways, and mechanical loading responses. Furthermore, reduced estrogen concentrations resulting from LEA can enhance osteoclast activity, leading to excessive bone resorption [4].

Carbohydrate intake predicts quick hormonal responses to stress and improves explosion responses during exercise when above 5.0 g/kg/day, higher carbohydrate intake stimulates chronic growth hormone release (despite its acute suppressive effects); together, carbohydrate and protein intake predicted the late prolactin response (30 min after hypoglycemia), muscle recovery speed was directly predicted by overall calorie intake, regardless of the proportion of macronutrients, protein intake prevents body and visceral fat accumulation and increases basal metabolism rate when above 1.6 g/kg/day, sleep patterns are the major determinants of mood states, and excessive concurrent physical and cognitive effort decreases fat oxidation, increases muscle catabolism, and impairs libido [19].

### 3.4. Psychological and Psychosocial Factors

Although the role of physical factors in OTS has been extensively studied, there is growing recognition of the importance of psychological factors in its development among athletes. Psychosocial factors have also gained attention as potential contributors to both OTS and bone stress injuries among athletes. Psychological stressors associated with high-performance sports may impact an athlete’s risk of these conditions through various mechanisms, including altered immune function, disrupted sleep patterns, or maladaptive coping strategies [6]. OTS is a complex condition that arises from an imbalance between training load, nutrition, and recovery time. It is characterized by a decrease in training performance and persistent fatigue, which can have detrimental effects on an athlete’s physical and mental well-being. This section aims to dive into the physiological changes associated with OTS by incorporating additional research studies [1].

Psychological stressors have been identified as crucial contributors to the onset and progression of OTS. These stressors can arise from multiple sources such as training demands, competition pressure, personal life stressors, and perfectionistic tendencies. The review highlights how these stressors can lead to increased levels of anxiety and depression symptoms among athletes with OTS. However, it is important to note that not all athletes who experience high levels of psychological distress develop OTS. This suggests that individual differences play a role in determining susceptibility to OTS [1].

To gain a deeper understanding of the relationship between psychological factors and OTS, recent research has proposed approaching OTS as a complex system phenomenon. This perspective acknowledges the intricate interactions between various biological systems involved in OTS development. Authors have suggested employing techniques like transomics analyses and machine learning for comprehensive evaluation of individuals with suspected or diagnosed OTS. They have also highlighted that future research should focus on the analysis of brain neural networks in relation to the prevention and management of OTS. Neuroimaging studies could provide information on how prolonged exposure to psychological stress affects brain structure and function among athletes with, or at risk of developing, OTS. Furthermore, investigating hypothalamic–pituitary–adrenal responses to stress may elucidate hormonal imbalances associated with excessive training loads and inadequate recovery periods in athletes prone to developing OTS. Although psychological interventions have shown promise in managing various mental health conditions among athletes, their effectiveness specifically in preventing or managing OTS remains an area that needs further exploration. Valovich McLeod et al. suggest that cognitive behavior therapy (CBT) and stress management techniques could be valuable approaches to address psychological distress associated with OTS [20].

As pointed out by Maccagnano at al., for shoulder arthroplasty it is very important to perform a psychological analysis of each patient in order to choose the appropriate treatment [21]. This rule can also be applied to injured paralympic athletes.

Psychological factors play a significant role in the development of overtraining syndrome among athletes. Psychological stressors arising from training demands, competition pressure, and stressors of personal life can contribute to increased levels of anxiety and depression symptoms among individuals with OTS. Recent research suggests approaching OTS as a complex system phenomenon that involves interactions between multiple biological systems.

### 3.5. Hormonal Status, Oxidative Stress, and Immune System

Hormonal imbalances play an important role in the pathophysiology of OTS. Cadegiani and Kater conducted a study investigating the predictive value of basal hormones in male athletes with OTS. Their findings revealed lower levels of testosterone and higher levels of estradiol in athletes with OTS compared to healthy individuals. These hormonal alterations may contribute to the fatigue and decreased performance observed in OTS [22].

Immune system dysfunction has been identified as a contributing factor to both OTS and bone stress injuries. Schwellnus et al. discussed the relationship between the training load in sports and the risk of illness and overtraining. They highlighted that excessive training load can lead to immunosuppression, making athletes more susceptible to infections and other immune-related disorders. This compromised immune function may further exacerbate fatigue symptoms and impair the ability of an athlete to recover [3].

Collectively, exploring the physiology and mechanisms underlying overtraining syndrome is crucial to unraveling its complexities. Hormonal imbalances such as altered testosterone–estradiol ratios have been observed in individuals with OTS. Oxidative stress resulting from the production of reactive oxygen species in exercise can contribute to fatigue symptoms seen in athletes with OTS. Inflammation and immune system dysfunction also play a significant role in both overtraining syndrome and bone stress injuries among athletes.

## 4. Diagnostic Approaches to Overtraining Syndrome and Bone Stress Injuries

The accurate and timely diagnosis of OTS and bone stress injuries is crucial for effective management and prevention of long-term complications among athletes. This section aims to critically review current diagnostic methods for OTS and bone stress injuries, considering additional research from various articles.

**Figure 1:**
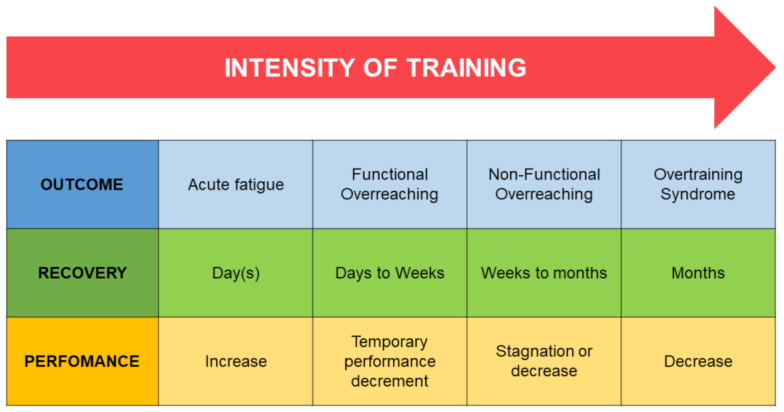
Differences between overreaching and overtraining [23].

Differences between OTS, functional and non-functional overreaching, that are crucial in an appropriate approach to diagnosing OTS are shown in Figure 1.

The diagnosis of OTS involves a combination of subjective measures, such as questionnaires and symptoms checklists, along with objective biomarkers, including hormone levels and inflammatory markers [24,25]. Subjective measures provide information on an athlete’s perception of their training load, fatigue, mood states, recovery status, and overall well-being. Various validated questionnaires have been developed to assess different aspects related to OTS. For example, the Recovery-Stress Questionnaire for Athletes (RESTQ-Sport) evaluates an athlete’s balance between recovery demands and stressors [24]. On the other hand, the Profile of Mood States (POMSs) assesses various mood dimensions that may be affected by overreaching or excessive training loads [26].

Objective biomarkers offer physiological insights into an athlete’s response to training load and recovery status. Hormonal imbalances have been observed in athletes with overtraining syndrome; decreased testosterone levels and increased estradiol levels are commonly reported findings. Furthermore, alterations in cortisol secretion patterns have been associated with the development of OTS. However, it is important to note that hormonal changes can be influenced by factors such as age, sex, phase of the menstrual cycle in women, time of day when samples were collected, individual variations in hormonal responses to exercise stressors, or other factors unrelated to the OTS itself [25].

Inflammatory markers also play a role in the diagnosis of OTS. Studies have reported elevated levels of C-reactive protein (CRP) and interleukin-6 (IL-6) in athletes experiencing OTS [25]. These markers reflect the systemic inflammatory response to excessive training loads, indicating a potential link between chronic inflammation and the development of OTS. However, it is important to interpret these findings with caution as exercise-induced inflammation can also occur in response to acute bouts of intense exercise, without necessarily indicating the presence of OTS.

Despite the progress made in diagnostic approaches for OTS, there are still limitations that need to be addressed. Subjective measures are prone to individual interpretation and reporting bias. Athletes may underreport symptoms due to fear of negative consequences, desire to continue training, or lack of awareness of the severity of their condition [24]. Additionally, subjective measures are heavily based on self-reporting, which can introduce variability into the assessment process.

Objective biomarkers show promise but require further validation and standardization for clinical use. Hormonal changes observed in athletes with OTS may not be specific enough for accurate diagnosis as hormonal fluctuations can occur due to various factors other than overtraining alone [25]. Similarly, inflammatory markers are influenced by multiple factors, including acute bouts of exercise or infection/inflammation unrelated to OTS itself.

Additionally, advances in technology offer opportunities for real-time monitoring of training load and recovery status using wearable devices or mobile applications [3]. These technologies can provide objective data on training volume, intensity, heart rate variability, sleep quality, and other relevant factors to aid in the diagnosis of OTS. Integrating these technological advances with subjective and objective measures can improve diagnostic accuracy and facilitate early intervention.

Collectively, diagnosing OTS requires a multifaceted approach that combines subjective measures and objective biomarkers. Subjective measures, such as questionnaires, provide insights into an athlete’s perception of their training load and well-being. Objective biomarkers offer physiological information but require further validation for clinical use. Future research should focus on integrating multiple biomarkers with advanced technology to enhance diagnostic accuracy and facilitate timely intervention for athletes at risk of developing OTS.

## 5. Prevention and Management Strategies for OTS and Bone Stress Injuries

Understanding and implementing effective prevention and management strategies for OTS and bone stress injuries is crucial to optimizing athletes’ health and performance. This section will review current strategies for preventing overtraining syndrome among athletes, the role of strength training and conditioning programs in reducing the risk of bone stress injuries and explore treatment options for both overtraining syndrome and bone stress injuries.

To prevent overtraining syndrome among athletes, various strategies have been proposed. One approach is periodization of training, which involves planned variations in training volume and intensity to optimize performance while minimizing the risk of overtraining [1]. By carefully manipulating training variables such as load, frequency, duration, and recovery periods throughout different phases of a training program, coaches can ensure that athletes achieve optimal adaptations without exceeding their recovery capacity. Periodization has been shown to improve athletic performance in various sports by balancing workload with adequate rest [27].

In addition to periodization, monitoring biomarkers during preseason training may help identify early signs of overreaching or overtraining. A study by Clemente et al. investigated hematological and biochemical markers in professional soccer players during the preseason period. The results showed an increase in platelet levels, but decreased absolute neutrophil counts, absolute monocyte counts, and calcium levels after preseason training. Furthermore, there were significant increases in creatinine, alkaline phosphatase, C-reactive protein, cortisol, and testosterone levels. Monitoring these blood measurements could provide valuable insight into an athlete’s physiological response to changes in training load [28].

In terms of bone stress injury prevention, strength training and conditioning programs play a crucial role. Resistance training has been shown to improve sport performance, improve body composition, and reduce the rate of sport-related injuries. By incorporating exercises that target specific muscle groups and movements relevant to the sport of the athlete, strength training helps improve biomechanics and reduce the risk of overuse injuries [1].

Furthermore, nutritional support plays a vital role in both preventing OTS and promoting recovery from bone stress injuries. Adequate energy intake is crucial to meet energy demands during intense training periods [3]. Low energy availability can lead to relative energy deficiency in sport (RED-S), which has severe health consequences if not addressed properly [4]. Adequate intake of macronutrients (carbohydrates, proteins, fats) and micronutrients (vitamins, minerals) is crucial to meet the metabolic demands of exercise and promote recovery processes. Athletes should work with sports nutrition professionals to ensure that they meet their nutritional needs based on their activity levels.

When OTS occurs despite preventive measures, appropriate treatment strategies are essential for recovery. Rest is crucial to allow the body to recover from the accumulated fatigue and stress associated with OTS [28]. Rehabilitation protocols should focus on gradually reintroducing training while considering individual responses to treatment. Physical therapy interventions such as manual therapy techniques, therapeutic exercises tailored to specific needs, and modalities can help promote healing, restore function, and prevent future injuries [20]. Medications such as non-steroidal anti-inflammatory drugs can help manage pain and inflammation associated with bone stress injuries [2]. However, it is essential to consider possible side effects and consult with healthcare professionals before using pharmacological interventions.

Participation in physical training can be highly skeletally demanding, particularly during periods of rapid growth in adolescence, and when competition and training demands are heaviest. Sports involving running and jumping are associated with a higher incidence of bone stress injuries and some athletes appear to be more susceptible than others. Maintaining a very lean physique in aesthetic sports (gymnastics, figure skating and ballet) or a prolonged negative energy balance in extreme endurance events (long distance running and triathlon) may compound the risk of bone stress injuries with repetitive mechanical loading of bone, due to the additional negative effects of hormonal disturbances [29].

Finally, effective prevention and management strategies for OTS and bone stress injuries require a comprehensive approach that includes periodic training, monitoring biomarkers during training periods, incorporating strength training programs, ensuring adequate nutritional support, promoting rest, and rehabilitation protocols when needed. By implementing these strategies in a customized manner while considering personalized athlete characteristics, coaches, sports medicine professionals, and athletes themselves can optimize performance outcomes while minimizing the risk of OTS and subsequent bone stress injuries in athletic populations. More research is needed to explore additional preventive measures, as well as to refine existing strategies to improve athlete health and performance.

## 6. Limitation, Strength, and Future Aspects

The relationship between OTS and bone stress injuries among athletes has been extensively studied, but there are still several areas that require further research to enhance our understanding of this complex relationship. This section will critically review the literature and identify key research gaps, highlighting the need for additional investigations in specific populations and aspects of OTS and bone stress injuries.

An area that requires further research is the identification of specific risk factors and mechanisms underlying the development of OTS and its association with bone stress injuries. Although some intrinsic and extrinsic risk factors have been identified, such as age, sex, training volume/intensity, inadequate recovery periods, there is a need for more comprehensive studies that consider multiple factors simultaneously. For example, a study by Matos et al. investigated various potential risk factors for overuse injuries in young athletes aged 12–17 years. They found that LEA, menstrual dysfunction in female athletes, a previous history of injuries, low body mass index, and high intensity of training load were significant predictors of developing overuse injuries [1].

Furthermore, it is essential to explore the impact of psychological stressors on the development of OTS and subsequent bone stress injuries among athletes. Psychological factors play a crucial role in an athlete’s overall well-being and performance. A study by Costa et al. examined the relationship between psychosocial variables (e.g., perceived stress levels) and bone health outcomes in elite female artistic gymnasts. The findings highlighted how psychosocial factors can influence hormonal balance, nutritional status, and energy availability, ultimately affecting bone health outcomes [2].

More research is needed to understand the long-term consequences of OTS on bone health outcomes among athletes. Longitudinal studies that evaluate changes in bone mineral density, bone turnover markers, and fracture risk over extended periods can provide valuable information on the recovery process and long-term effects of OTS on bone health. A study by Barrack et al. followed a group of male endurance athletes for two years to assess changes in BMD and incidence of stress fractures. The findings revealed that LEA was associated with decreased bone mineral density and increased risk of stress fractures [30].

Also, research should focus on specific populations that may be more susceptible to OTS and bone stress injuries. For example, youth athletes have unique physiological characteristics that may influence their response to training and injury [1]. Understanding the specific needs and vulnerabilities of these populations will help tailor prevention and management strategies accordingly. Studies are necessary to examine the impact of gender-specific factors on the development of overtraining syndrome among female athletes [31]. Female athletes face distinct challenges related to menstrual status, energy availability, and hormonal fluctuations, which may contribute to their increased susceptibility to both OTS and bone stress injuries.

In addition, there is a need for standardized diagnostic criteria and objective measures that can accurately identify overtraining syndrome from other conditions with similar symptoms. Currently, diagnosis is based on subjective measures such as questionnaires or checklists of symptoms combined with objective biomarkers such as hormone levels or inflammatory markers [20,22]. However, more research is needed to validate these diagnostic approaches against gold standard methods while considering individual variations in response to training load.

In terms of prevention strategies for OTS and bone stress injuries, future research should investigate the effectiveness of targeted interventions beyond traditional approaches, such as training periodization or adequate recovery periods. For example, studies exploring the potential benefits of psychological interventions, including mindfulness-based training or cognitive behavioral therapy, could provide valuable information on the management of psychological stressors and the reduction in the risk of developing OTS.

Lastly, research should explore novel methodologies to assess bone health and injury risk in athletes with overtraining syndrome. Advanced imaging techniques, such as magnetic resonance imaging, can offer more accurate evaluations of bone microarchitecture and early detection of stress fractures [22]. Incorporating biomechanical analyses, such as gait analysis or motion capture systems, could provide further insight into the movement patterns and loading mechanics that contribute to bone stress injuries.

While significant progress has been made in understanding the relationship between OTS and bone stress injuries among athletes, there are still several areas that require further investigation. Future research should focus on identifying specific risk factors and mechanisms underlying these conditions, exploring the impact of psychological stressors, understanding long-term consequences on bone health outcomes, investigating the vulnerabilities of specific populations, establishing standardized diagnostic criteria, evaluating targeted prevention strategies beyond traditional approaches, and exploring novel methodologies for assessing bone health and injury risk. Addressing these research gaps will improve our understanding of this complex relationship and improve the prevention and management of overtraining syndrome and bone stress injuries among athletes. This paper could be useful as a step for a consensus on pathology in other clinical areas [32].

## 7. Conclusions

In conclusion, various intrinsic and extrinsic factors contribute to an athlete’s susceptibility to developing OTS. Age, sex, genetics, previous injury history, training volume/intensity, inadequate recovery periods, and nutritional deficiencies or imbalances all play a role in increasing the risk of OTS among athletes. Understanding these risk factors is crucial for implementing appropriate preventive strategies tailored to individual athletes’ needs. Further research should focus on elucidating the underlying mechanisms through which these risk factors influence the development of OTS while considering their potential interaction with other variables, such as psychological factors. In general, a comprehensive approach that combines physical and psychological evaluations, together with individualized training programs and support systems, is necessary to effectively prevent and manage OTS among athletes.

## Data Availability

Data used for analysis are contained within the article.
